# The ghostwriter and the test-tube baby: a medical breakthrough story

**DOI:** 10.1017/mdh.2025.10025

**Published:** 2025-07

**Authors:** Nick Hopwood

**Affiliations:** Department of History and Philosophy of Science, University of Cambridge, Cambridge, United Kingdom

**Keywords:** Autobiography, Ghostwriting, Human embryos, Infertility, In vitro fertilisation (IVF), Women in science and medicine

## Abstract

Ghostwriting autobiographies has gained so high a profile that novels and films focus on the ghost. To deepen understanding of such collaborations in science and medicine, this article reconstructs the making of *A Matter of Life* (1980), ‘the sensational story of the world’s first test-tube baby’. Although critiqued by feminist scholars, revised through research and embellished in fiction, this double autobiography of Robert Edwards and Patrick Steptoe is still the standard history of the British team’s work to achieve in vitro fertilisation (IVF). It is thus high time to investigate the debt acknowledged only by ‘gratitude for his invaluable help’ to the physician and poet Dannie Abse. I use previously unexploited manuscripts to illuminate relationships among authors, rewriter, and editor, and among those they cast as involved in the research. The records show that Abse rewrote underwhelming drafts for a publisher that had bought and sold the doctors’ story of the ‘baby of the century’ and needed a bestseller. To engage readers, he reworked the text so that alleviating infertility appeared as a career-long quest. As a result of adding vivid scenes with characters and expository dialogue, Abse began to give women—wives, assistants and patients—larger roles in the drama. The objections of Edwards and his circle to various literary references and factual claims were overruled. Yet the authors came across more sympathetically, and IVF was promoted more effectively, than in their own drafts. The process puts recent retellings of the story into perspective and exemplifies how collaboration can shape scientific and medical autobiographies.

Biography is the dominant mode for stories about science and medicine, and autobiography stakes a special claim to authenticity.^1^ The film *Joy* (Netflix, 2024) has brought new audiences to the project that produced the first baby born after in vitro fertilisation (IVF). The screenwriters deployed fresh findings to amplify the crucial role of technician Jean Purdy and to pay more attention to the disappointed volunteers.^2^ But they took as their ‘base’ the joint autobiography of the architects of IVF, geneticist Robert Edwards and gynaecologist Patrick Steptoe.^3^ Thanks to the depth of detail and the authors’ authority, *A Matter of Life: The Story of a Medical Breakthrough* (1980) remains the default account. Thirty years ago, the literary scholar Susan Squier critiqued this tale of two male heroes producing a pregnancy, but popular histories still reproduced excerpts, and research has only begun to revise the narrative.^4^ Here, I explore the genesis of the story for the first time. I reveal that much of what has been ascribed to Edwards and Steptoe is the work of a ghostwriter whose hitherto unexamined manuscripts provide a rare opportunity to explore how collaboration on autobiographies has moulded histories of science and medicine.

Ghostwriting has become a feature of celebrity autobiography and literary explorations of identity and storytelling. Today, there is little shame in employing a ghost, only in concealing the fact. True, the boundaries between revising, commenting, editing and ghosting are fluid. Nor in some ways is a ghosted autobiography different from any other; to write one’s life has been compared to acting as one’s own ghostwriter.^5^ Memoirs are all at some level ‘fictions’, ‘shaped by … generic expectations’ and using ‘literary structures and tropes to rewrite … experience to fit … conventions’.^6^ But this form of collaborative writing is distinctive—and varied—as manuals, memoirs, novels and histories have shown.^7^ Historian John Hill Burton played to Victorian stereotypes of an uncivilised Africa in revising John Hanning Speke’s ‘atrocious’ writing into the *Journal of the Discovery of the Source of the Nile.* ^8^ Journalist Alex Haley co-authored the ‘as told to’ *Autobiography of Malcolm X* to an extent that it is debated whether ‘ghostwriter’ does justice to his contribution.^9^

Edwards and Steptoe also had a well-known book doctor—Dannie Abse was the most successful medical poet of his generation and his own celebrated autobiographer—and his ‘ghostwriting’ papers, supplemented with other manuscripts, offer extraordinary access. We have evidence from the authors’ first drafts through interviews, rewriting, and correction—by Edwards, his wife and fellow scientist Ruth Edwards née Fowler and Purdy—to publication, reviewing and eventual revision.^10^ Histories will need to take account especially of the drafts and notebooks, near-contemporary records that contain new details and versions of events. My purpose here is to reconstruct how a rewriter’s participation changed the process and product, and so to bring insights from studies of collaborative autobiography into science and medicine while highlighting the specificities of ghosting scientific and medical lives.^11^

The process gave the ghost more power than one might expect. Celebrities are often taken to have the upper hand in collaborative autobiographies—when they care to read the results—and it is regarded as ethical for an author to have editing rights.^12^ The demand for technical accuracy might be assumed to let scientists and physicians call even more shots. But here the publisher’s editor insisted that a writer adapt the authors’ rough drafts into a quick, profitable bestseller that would also more sympathetically press the case for IVF. Abse did not just practise a kind of ventriloquism; sometimes, like the Ghost in Robert Harris’s novel of the same name, he drew on his own experiences to craft for his clients ‘lives they never even realised they had’.^13^ Abse’s interviews improved and enriched the drafts, but Edwards’s and his associates’ corrections were often overruled.

The collaboration of authors, editor and rewriter thus formed the product. In other words, *A Matter of Life* is different from James Watson’s *The Double Helix*, the book to which it was most often compared, not just in being a double autobiography, such as Watson and Francis Crick famously did not produce, or in straddling science and medicine, but also because a ghost reworked the tale.^14^ Abse’s preoccupations and literary skills supplied most of the cultural references that Squier identified—without considering his involvement—as giving the book its ‘metaphoric depth and richness’.^15^ He made Edwards’s and Steptoe’s texts conform more fully to expectations of scientific origin stories and medical dramas, notably by imposing a more consistent quest to overcome infertility, a condition the authors were working to raise in public and biomedical priorities. By expanding and fleshing out the cast he granted women, including Purdy and the principals’ wives, more of a role. He ensured an effective introduction of the star patient, Lesley Brown. This distribution of credit handed the authors more chances to acknowledge female help, but was driven by the demand for human interest through novelistic detail, expository dialogue and characterisation, not feminism.

It testifies to the hidden power of ghostwriting in medical and scientific autobiography that a book published forty-five years ago still provides the baseline history of the science and medicine behind IVF. In many ways, and not least in efforts to make women’s contributions more visible, storytellers continue to rely on, extend and revise what is as much Abse’s work as Edwards and Steptoe’s. Reconstructing its genesis shows how the collaborators’ interests and relations shaped and continue to shape the representation of those undertaking the scientific and medical work. Now that scientists and physicians routinely co-author autobiographies, such cooperation is more widely relevant than ever.^16^

## Bringing in a Writer

Medicine joined the cult of celebrity of the 1960s. English broadsheets treated an instant autobiography of the heart-transplant surgeon Christiaan Barnard as a sad symptom of fame. *The Times* blamed the co-writer, a US journalist, for ‘jangling emotionalism’ but lamented that because ‘this spurious work … is written … in the first person … one receives the powerful impression that a man has … succeeded in libelling himself’. In *The Observer*, a critic of the surgery (which patients did not then survive long) deplored the book as ‘one of those packaged products … in which a famous person is presented … in a predigested form, ready for instant use by the media’. Sure enough, a tabloid agony aunt judged it ‘riveting’.^17^

A decade later, the ‘baby of the century’ made a huge global news story and, although controversial, a happier one. Robert Edwards of Cambridge, a distinguished reproductive scientist who had learned to fertilise human eggs in dishes, collaborated with Patrick Steptoe of Oldham near Manchester, the respected British pioneer of laparoscopy, or keyhole surgery for accessing the female reproductive system. Since their partnership went public in 1969, Edwards and Steptoe had been denied Medical Research Council (MRC) funding and vilified by certain influential biologists and medics, not to mention journalists and theologians. But, as infertility rose up the agenda, the prospect of bypassing blocked Fallopian tubes using in vitro fertilisation and transfer of the resulting embryo to the uterus was exciting.^18^

After long years of trying, involving at least 282 infertile women volunteers, Steptoe finally obtained an apparently normal pregnancy for one of them, Lesley Brown. In April 1978, the press broke the story, and reporters besieged the hospital. Steptoe attempted to control things by offering exclusive access to the parents at an auction won by the *Daily Mail.* This chequebook journalism further tarnished his and Edwards’s reputations, and they were accused of withholding information from their peers. They did stage a press conference on 26 July 1978, the morning after the birth, but two-thirds of a column in *The Lancet* three weeks later gave little away. Following a sympathetic TV documentary broadcast that August, they provided the first major disclosure at a symposium after a second birth in January 1979. This triumphant event satisfied many experts but by no means all competitors, and the full journal articles did not appear till September 1980.^19^ By then, their autobiography had been out for nearly half a year.

Steptoe and Edwards wanted to tell their side of the story, while settling scores with critics, and the sooner the better, also in commercial terms. But they had rejected ‘enormous sums’ from newspapers, magazines and TV networks because these demanded an ‘unacceptable’ exclusivity.^20^ I do not know how they chose the prominent London publisher Hutchinson, whose list ranged from middlebrow to academic, but presumably prestige and independence trumped profits. The deputy managing editor, Harold Harris, pulled off this coup. A former journalist and literary editor of the London *Evening Standard*, most interested in non-fiction, he had also recruited the bestselling novelist Frederick Forsyth.^21^ Eight days after the birth, Harris confirmed an advance of £60,000 (equivalent to about £400,000 in 2025) for ‘world volume and serial rights’.^22^ A month later, in September 1978, a contract was signed.^23^

In October, author biographies, a proposal and a sample (which Harris edited from Steptoe’s draft) went to the Frankfurt Book Fair, where Harris sold foreign rights. The ‘Book on the first test-tube baby’ would tell of careers that led to a ‘chance meeting’; then of how the authors’ work resulted in the ‘birth … that hit the headlines and television screens of the world’. Hutchinson promised ‘the story of two men of purpose and determination … a story … of hope and despair, of crushing disappointments, and dramatic discoveries … criticism and even hostility’, culminating in ‘the enormously poignant moment when Patrick Steptoe himself handed the lusty, healthy infant … to a mother who had been told … that she could never have a baby’.^24^ The firm had a great week in Frankfurt, selling rights for a ‘hat-trick’ of books: this one, a new Forsyth novel and the autobiography of sailor Naomi James.^25^

Edwards and Steptoe delivered the remaining drafts between January and early March 1979.^26^ The working-class Yorkshireman and the patrician pianist would each describe ‘his own part’.^27^ Having retired from the National Health Service, Steptoe had time, but Edwards was busy setting up a clinic, writing the thousand-page monograph *Conception in the Human Female* and moving house. Steptoe took charge of the birth, but Edwards drove the project and had lived with it longer. The book cemented this dominance when he agreed to write two-thirds: an early table of contents gave him three and Steptoe two chapters before they met, then him five and Steptoe two on their collaboration (Table 1).^28^

**Table 1.** Chapters of *A Matter of Life* from draft to book. The right-hand column gives the first-edition chapter titles, columns to the left as far as possible those of the corresponding authors’ drafts (by RE and PS) and rewrites and revisions by Abse and Harris (DA and HH), reordered to match the book. Read left to right for the process of revision, right to left to find drafts by folder and file number, all in the Dannie Abse Papers, National Library of Wales, unless noted as being in the Robert Edwards Papers, Churchill Archives Centre. Numbers are as in the column headings and authors as in the left-hand column unless given in a cell. ‘Bob’s corrections’ (folder 280) is the most complete typescript and includes corrections from his circle. The copies in folders 281 and 283 are similar but less complete. Folder 282 contains the late rewrite of Chapter 1. ‘TOC’ adds titles from folder 277, ‘Contents’, where different from those in the drafts.

	Synopsis and sample chapters			RE and PS drafts (277)	‘DA’s revisions’ (278–9)		‘Bob’s corrections’ (280)	Book
HH	Book on the first test-tube baby 12.9.78 (EDWS 4/14/1)							
PS				RE, Chapter One ‘Very tentative draft indeed’, 27.2.79	DA’s drafts leading up to PS, 1 The Quest (278/5–9)		1 The Quest DA’s rewrite (282/1–6) PS concert (307/10/2–4)	1 The Quest
RE				Chapter Two: Bob ‘1st draft’, 31.1.79	2 The Second Chance (278/11–14)		2 The Second Chance	2 The Second Chance
RE	Copy of Edinburgh: The Beginnings of an Idea <3 The Mouse House>, with edits (by HH?) (277)		3 The Mouse House, edited (277)	Chapter Three: Edinburgh: The Beginnings of an Idea ‘1st draft’, 31.1.79	3 The Mouse House (278/17–19)		3 The Mouse House	3 The Mouse House
					4 Night into Day (278/21)		4 Night into Day	4 Night into Day
RE	Copy of Chapter 4: The Topsy-Turvy Years, partly edited (by HH?) (277)			Chapter Four: The Topsy-Turvy Years ‘1st draft’, 31.1.79	5 The Control Dish (278/22)		5 The Control Dish	5 The Control Dish
RE					6 The Green Chromosomes (278/23)		6 The Green Chromosomes	6 The Green Chromosomes
RE					7 The Path Road Taken (278/24–6)		7 The Road Taken	7 The Road Taken
RE							8 Eureka	8 Eureka
				Chapter Seven: Meeting of the Minds ‘1st draft’, 31.1.79				
PS				Steptoe – Early Years to End of War: Chap. 5 [TOC: Patrick] 9.1.79	9 To Oldham then I Came (278/27)		9 To Oldham then I Came	9 To Oldham then I Came
PS					10 If only (278/28–30, /32)		10 If only	10 If only
PS				Chap. 6 [TOC: Laparoscopy Comes of Age] 14.2.79	9 Inside the Abdomen (note of title in 278/3) 11 Laparos[c]opy Comes of Age (278/31)		11 Laparoscopy Comes of Age	11 Laparoscopy Comes of Age
RE				Chapter Eight: The Good Years ‘1st draft’, 31.1.79	11 The Magic Culture Fluid (278/20, /34–6, /38–40)		12 The Magic Culture Fluid	12 The Magic Culture Fluid
RE					12 Four Beautiful Human Blastocysts (278/33, /37)		13 Four Beautiful Human Blastocysts	13 Four Beautiful Human Blastocysts
RE				Chapter Seven <9>: The Difficult Years 23.2.79	14 The Difficult Years (279/1–2)	14 More Opposition The Wounds of Opposition (279/3)	14 The Wounds of Opposition	14 The Wounds of Opposition
RE						15 The Shindy in Washington (279/4)	15 The Shindy in Washington	15 The Shindy in Washington
RE					16 Bad Days in Oldham (279/12–14)		16 Bad Days in Oldham	16 Bad Days in Oldham
RE				Chapter Eight <10>: Wind of Change	(The) Wind of Change (279/5–7)		17 The Wind of Change	17 The Wind of Change
RE					18 (The Breakthrough?) Back to Nature (279/8–10)		18 Back to Nature	18 Back to Nature
PS				The Pregnancy [TOC: 12 A Difficult Pregnancy] (279/15)	[The Pregnancy] (279/16) 20 A Patient Called Lesley Brown (279/17, /19)		20 21 A Patient Called Lesley Brown	19 A Patient Called Lesley Brown
PS				Ch. 12 continued 6.3.79	21 22 Into the Test Tube (279/29)		22 Into the Test Tube	20 The Minuscule Dot of Life
RE				Chapter 11: The Breakthrough 27.2.79	19 The Breakthrough (279/11) PS, Patrick; RE, The Crucial Year (part of 279/28)		19 The Breakthrough	21 The Breakthrough
RE					20 More Pregnancies The Noise of the Day, the Peace of the Night (279/20–1) The Crucial Year (part of 279/28)		20 The Noise of the Day, the Peace of the Night	22 The Noise of the Day, the Peace of the Night
PS				Ch. 12 continued 6.3.79	23 The Delilah of the Press (279/18, /27, /30) RE, Epilogue Stuff (part of 279/28)		23 The Delilah of the Press	23 The Delilah of the Press
PS	Final Chapter ‘As taped’ [TOC: 13 Louise] Final Chapter <Ch. 13> (277)	Prologue, ed. HH (EDWS 4/14/1)			24 Final Chapter Day of Decision (279/24–5)		24 Day of Decision	24 Day of Decision
PS		Final chapter: Fulfilment, ed. HH (EDWS 4/14/1)			25 Birth of a Baby (279/25)		25 Birth of a Baby	25 Birth of a Baby
RE				[3 pp. on the future]	26 Farewell to Oldham (279/23, /26, /31–2)		26 Farewell to Oldham	26 Farewell to Oldham
PS				Epilogue [August 1978 – March 1979] (279/22)				

From an early stage, Harris reckoned that ‘something rather more than an editing job is going to be required’. Aspects needing attention included

the … structure …, the arrangement and pacing of the events and climaxes, the introduction of other characters, the explanation of technical terms where this is required, the over-riding necessity of making sure that the two narrators dove-tail into each other and, indeed, the desirability of ensuring that the two authors themselves come across to the reader in the way that they would wish.

He proposed that someone ‘re-write’ the text ‘for publication’. Edwards resisted, although ‘not fully satisfied myself’ with his drafts.^29^ Harris insisted that because ‘your narrative … moves on pretty remorselessly, all on the same level’, ‘the reader is cheated of … the excitement’ ‘impart[ed] to those who are fortunate enough to listen to you, thereby forfeiting the sympathy which they feel for your work and yourself’.^30^ This posed ‘a big problem because so much needs to be done to make it … bestseller material’. The manuscript was also ‘too short’ at around 60,000 words. Harris wanted another 30,000 ‘from (a) making things a bit easier for the lay reader … and (b) by writing in a rather more relaxed way with … some light and shade’. In sum, while ‘the material is absolutely fascinating’, ‘the presentation is not’.^31^

The revision, Harris told Edwards and Steptoe, ‘will need a writer of tact, imagination, skill and, preferably, one with some medical knowledge. … It would be tempting providence to suggest that such a paragon exists, but I do think we have available the best person for the job.’ Having already persuaded Dannie Abse in principle following lunch at London literary hangout Bertorelli’s, Harris introduced his book doctor as ‘a doctor of medicine … a novelist, playwright and … the outstanding serious poet on the Hutchinson list’. A full-time chest physician, Abse was becoming the leading medical poet since William Carlos Williams. ‘He wrote an autobiography in 1974 … he writes, under a pseudonym, a regular medical column in the *Sunday Express*’. Proposing a meeting, Harris spelled out that ‘“the third man”’ would need an advance of £5,000, borne by Hutchinson but raising the sum at which royalties began to be paid, and a ten per cent share of these (which would thus start for Edwards and Steptoe at a joint nine rather than ten per cent). Brandishing a financial incentive to go with the public-relations one, Harris was ‘confident that … your actual earnings in cash will be considerably greater with Dannie Abse’s assistance than without it’. The authorship could be expanded to ‘with (or in association with) Dannie Abse’, Harris suggested, but it never was.^32^

Harris convened an interview in March 1979.^33^ Steptoe struck Abse as a ‘stereotypical’ consultant, though ‘few [were] as innovative and bold as he’. ‘Edwards seemed to me something else. I had never met a pure scientist before.’ He was ‘without side, without a mask, teeming with ideas … [and] prepared to take risks that would make most doctors blink’. Abse ‘could not help but respond to his account of … the beginnings of life’, which Harris also praised as ‘most moving and delightfully done’. Having ‘in ludic mood’ ghosted, ‘in [Edwards’s] persona, a paragraph or two’, Abse took on the job as ‘a challenge, a sort of game’.^34^

There was, then, to be a third man or a fourth, if we include Harris, who oversaw the major revision that Abse undertook between March and August.^35^ How did Abse go about his task?

## Burying the Real Life

Ghostwriters often move into the business after failing to have novels published or as a sideline to journalism. Abse, by contrast, had authored a fictionalised autobiography to great acclaim and a more straightforward one.^36^ For him, ‘autobiography is … a kind of fiction’ that, ‘with all its approximate resemblances, buries the real life of the autobiographer’ who ‘courageously destroys his past experiences by naming them’. Yet the process, ‘despite inadvertently altering …, obliterating …, accentuating …, may ultimately not only give us pleasure but reveal to us more about the world we live in, and more about ourselves’.^37^

Abse’s writing drew on several dualities. A technical expert disturbed by the modern world, he tried ‘to reconcile’ medicine and poetry, or ‘white coat and purple coat’, as he searched for meaning, even faith, but did not expect to find it.^38^ The deepest dyad was being British and Jewish after the Holocaust. Abse was also Welsh, born in Cardiff and with a house in Ogmore-by-Sea, twenty-five miles to the west, but lived in London, between bourgeois Golders Green and bohemian Soho, and wrote in English. His recollections interwove personal and public events:

It was July 1934 … when fires started on the English heaths; and in the forest, terrible jaws of flame consumed the turf and the shrieking trees with their jagged yellow fangs. Even as Keith and I sunbathed at Barry Island, all day long elsewhere there was the great crashing of dead branches, and columns of black smoke sat in the windless blue-hot skies. Yes, that July began with the torture of burnt trees in halcyon English woods; Captain Roehm shot dead in Germany, Dr Dollfuss shot dead in Austria, and a man called Hitler screaming …. ‘We the English’—shouted [Oswald] Mosley—‘we the English are being throttled and strangled by the greasy fingers of alien financiers.’ And he was talking about Dad and Mam, Wilfred and Leo, me and Uncle Isidore.^39^

An eleven-year-old Dannie is reimagined by weaving reconstructed experiences—Keith had no real counterpart—together with what he might have heard on the radio and from his older brothers’ newspaper reading.

Abse’s ghostwriting tried for similar transformations at one remove. But autobiography was trickier when it buried others’ lives. Private and public were harder to interlace when the private was not his own. Abse adopted a range of strategies to push literary culture in and draw the authors out.

At the generic end of a spectrum, Abse used background research to locate the science in periods and places and introduced references to embed it in traditions. His own shared experiences, not Welshness or Jewishness, afforded more personal ways in. Edwards was a similar age and also on the Left. Steptoe, ten years older, had trained in and practised medicine. Most personally, Abse interviewed Edwards several times in Cambridge and Steptoe in London.^40^ The interviews elicited descriptions of people and settings, recollections of motivations and interactions, and extra anecdotes. Abse pinned down facts, checked his understanding and obtained sources.

In some respects, then, Abse approached what happened and what it meant to the authors more closely than had their restrained accounts. But the vivid style created this impression even where additions emerged from his preoccupations. The corrections show that some allusions struck Edwards as inappropriate and that they sometimes disagreed over what he had said. He mostly objected in vain. Abse may have enjoyed adopting a persona but was an established writer with a distinctive voice. He and the publisher had the last word.

In crafting the story, Abse deployed standard writers’ techniques. By doubling the number of chapters and reorganising events to give each one an engaging shape and evocative title, he developed themes. Working through a series of handwritten and then typed drafts, he omitted tedious detail while pulling the reader in. Above all, he used the interviews to condense exposition into scenes with characters and dialogue.

## Birth Stories

Abse’s rewriting of Edwards’s chapters on his life from birth to postdoctoral fellowship will exemplify the approach. These took the most work because lab science offered less obvious human interest or relevance to infertility than clinical medicine. Though not quite ‘written in a style more fitted for a scientific paper’, Edwards gave minimal characterisation or dialogue and not much happened outside work—but he had included a few stories as well as topics that tempted with their glimpses of fascinatingly unfamiliar worlds.^41^

Edwards met the expectation that a subject’s character be foreshadowed by tracing back his ‘stubbornness and willingness for hard work’ to ‘an early age’. But, he admitted, ‘There was no indication of any scientific bent’. Only after paragraphs on his working-class childhood, schooldays and evacuation, including to a hill farm, did he explain the disaster of two years studying agriculture in Bangor by attributing his boredom to being ‘a scientist at heart’. Having switched to zoology, the lectures on fertilisation and embryology introduced him to ‘the study of the early stages of animal and human life’. He still left with a poor degree and felt fortunate to have been saved by acceptance onto a diploma course and then for a PhD at the Institute of Animal Genetics in Edinburgh.^42^

Narratively, Abse developed the trope of rescue. Renaming the chapter ‘The Second Chance’, he reordered the blow-by-blow account to open with Edwards’s mistake and move to zoology, too late for honours. Abse dramatised the ex-serviceman’s other struggle, against penury, as a far cry from the world Edwards had glimpsed in the officers’ mess, which Abse knew from the Royal Air Force.^43^

Thematically, Abse introduced readers to reproduction by arriving in the first paragraph, via crop seeds, at animal seeds. He pressed Edwards, about his year on the farm, for ‘Birth of sheep, pigs, foals, calves. Birth stories’. Abse noted nothing specific in reply but opened the next paragraph, ‘I had long been interested in the scientific processes of reproduction.’ He had Edwards recall ‘the natural laboratory behind hedges, wooden gates, byre and barn doors’ where ‘I had watched with wonder the birth of calves, sheep, pigs, foals as the aeroplanes of war droned on … overhead’.^44^ Abse traced a progression to the ‘more complicated questions’ that Edwards found ‘fascinating’ on his Zoology course. Edwards had written, ‘I was curious to know how spermatozoa reached the egg, why only one entered it, and how the embryo began its growth.’ Abse recast this as ‘On one occasion in the Zoology lab I looked up from a microscope thinking: *Why does only one spermat[o]zo[on] enter an egg?*’^45^ When shown the revised manuscript, Edwards demurred, ‘Not true’. Abse countered, ‘But Bob told me it was!’ and won.^46^

That question let Abse bring in Aldous Huxley’s ‘Fifth Philosopher’s Song’ which began with ‘A million, million spermatozoa’ and ended here with the narrator as sole survivor (‘the One was Me’).^47^ In a draft introduction to the autobiography, Edwards had mentioned ‘*Brave New World*, the spine-chilling essay’ with ‘its tone of despair, fear and frustration’.^48^ Abse’s more positive Huxley reference chimed with the theme of lucky survival but not yet: ‘When the summer examinations arrived the One that was Me did not do well.’^49^

Abse evoked a stronger sense of place, though the prose is uneven and purple passages intrude:

Autumn comes early to Edinburgh. In the evenings, when sudden lights in lofty freestone houses and in elegant shops paradoxically darken the city, the wind rises cold and fierce. It hustles the rusting leaves of the public gardens adjacent to Princes Street towards a premature oblivion. But I liked Edinburgh in October. I came to like it in every season, even when those chilling sea-mists crept in from the Forth. More importantly, I liked the work I was doing. … ‘A man’s character is his destiny’, wrote the Greek philosopher Heraclitus, two and a half thousand years ago. To a large degree, a man’s character is determined by his genes, and though I did not know it for certain then my own destiny was being determined in that Institute of Animal Genetics.^50^

We hear the poet’s voice, his sense of ‘the right word’ as ‘both surprising and just’, and the repeated ‘I liked’, a favourite transition device, but also observations close to cliché and a claim about destiny that moves, without noticing the contradiction, from genetic to environmental determination.^51^

Abse reconstructed Edwards’s doctoral and postdoctoral years as he split and lightened the draft ‘Edinburgh: The Beginnings of an Idea’. Picking up the already vivid description of nocturnal labour in the animal facility, Abse made ‘The Mouse House’ the theme of one smaller chapter and ‘Night into Day’, Edwards’s work with Ruth Fowler to stimulate ovulation in adult mice, the main business of the next.^52^ Abse researched more political, cultural and sporting information than he used, including details of plays that Edwards could not afford to see.^53^ Material about his politics fell by the wayside, too.^54^

Abse deployed research on the city to elaborate a story, elicited during an interview, that epitomised Edwards’s scientific approach in a relatable way and met expectations of competition between science and religion. A discussion of the night work led to his wondering about Life and Christianity:

My religious background had hardly been fervent … but now I began to attend experimentally the different churches in Edinburgh. I tried them all—the Presbyterian, the Episcopal, the Free Church of Scotland, the Methodist, the Baptist, the Congregational, the Lutheran, the Catholic, the Plymouth Brethren, the Quakers. I lingered in churches with stained glass windows and in those which boasted only plain glass. I looked up at hammer-beam roofs, at those with medieval vaulting and those with plain ceilings. … I suppose … I was on a church crawl …. However I did not become God-intoxicated. I felt eventually that the numinous and the mysterious could be found rather in the laboratory where each night I peered through a microscope at primitive sex cells.

Edwards objected in vain only to the word ‘numinous’—arousing religious emotion—‘What on earth is this?’^55^ While rejecting organised religion, Abse the poet ‘fulfill[ed] spiritual needs by articulating … mysteries and truths’.^56^

Abse’s ‘intertextualities’, most obviously the use of Huxley, anchored the account.^57^ But establishing Edwards’s interest in reproduction was not enough. Abse worked to focus this on human infertility.

## The Quest to Help the Infertile

Medical autobiographies cast heroic doctors in struggles against feared scourges. Here, the drama, and support for IVF, depended on creating awareness of the distress caused by infertility and presenting research on human embryos as producing healthy, wanted children. The chief narrative challenge in the first half of the book was that the birth of a baby became a joint goal only in the second half. In the mid-1960s, Edwards was still best known for immunological work directed at contraception. Even his early research into in vitro fertilisation aimed to understand genetic disease.^58^ Steptoe’s laparoscopy was applied mainly to sterilisation, and he performed many abortions. The authors had not hidden but did downplay these aspects of reproductive control. Abse exaggerated the teleology to establish the relief of infertility as their ambition from near the start. This would enlist readers, make the narrative more coherent and help to legitimise IVF.

Edwards’s youthful passion for agriculture encompassed animal breeding, and Abse managed to bring in ‘genetic engineering … so that we can talk early on about ethical propriety’.^59^ Edwards found this a stretch, and neither did he like Abse’s presentation of his PhD supervisor Alan Beatty’s key innovation as embryo transfer, which would be used in IVF, rather than insights into genetics.^60^ Edwards’s own draft ascribed his inspiration, after he joined the National Institute for Medical Research in 1958, to discoveries about human chromosomes and the potential to elucidate genetic disease.^61^

Edwards acknowledged a ‘more personal and domestic’ stimulus. ‘Ruth and I had started our family’ but had ‘less fortunate’ friends. Following an interview, Abse thickened this: ‘One couple whom we liked very much … wanted, but could not have, children. When they visited us that autumn and cuddled our babies I could not but be aware of the feelings aroused in them. The trees bore fruit, the clouds carried rain, and our friends, forever childless, played with our Caroline, our Jennifer.’ This led to the reflection, ‘I think it was their bearable but true predicament along with my preoccupation about ripening eggs and fertilization that made me wonder for the first time about the practicability o[f] replanting human embryos in the womb of a woman.’^62^

Edwards’s first success was ripening human eggs. Seeing the chromosomes under the microscope sparked ‘excitement beyond belief!’ Abse linked science to the clinic with reflections about ‘the possibility of helping people’ and ‘use one day to … childless couples such as our friends’. Edwards kept genetics in view by continuing the sentence ‘or to find the cause of Mongolism [Down syndrome]’.^63^ Abse pushed further by picking up Edwards’s comment that his supplier of human oocytes, gynaecologist Molly Rose, ‘delivered one of my daughters’. Abse learned that ‘the pregnancy was threatened’ but all turned out well and the girl was called Sarah. A paragraph riffed, with biblical quotation, on Abraham’s wife and the son she bore in old age. With progress in the lab, ‘The angel could come to Sarah.’ A lapsed Jew who had attended a Catholic secondary school, Abse valued religious literature. Edwards crossed the whole passage out—‘Danny [*sic*] This isn’t me!’—but it stayed.^64^ Abse then wrote as Edwards: ‘my primary preoccupation was what it had always been—to allow women, who were seemingly condemned … to a life of infertility, to bear their own … children fathered by their … husbands.’ This satisfied the desire for a consistent quest, but would it ring true, even with ‘to study human embryology’ put first?^65^

Helping patients was, by contrast, Steptoe’s day job, but he did not claim to have done much about infertility till the late 1950s. Abse worked to table the topic in Steptoe’s first substantive chapter by reducing the account of an Oxfordshire boyhood, organ and piano playing, studying then deciding on medicine, war service and two years as a prisoner of war in Italy to four printed pages. Focusing on a meal and musical evening with Kathleen Harding, the London consultant who had changed Steptoe’s attitude to infertility, Abse condensed his past life into memories prompted by his imminent departure for Oldham.^66^ The next two chapters gained purpose from Steptoe’s desire, for various reasons, including diagnosing and treating infertility, to see women’s reproductive systems not by laparotomy, an open operation, but through the keyhole by laparoscopy.

In the book, Edwards’s and Steptoe’s individual trajectories follow a first chapter that sets up ‘The Quest’ but was finalised last because it proved hard to get right. Harris had begun by splitting Steptoe’s sample final chapter, about the day of the birth, with the idea of sandwiching the rest in the middle (Table 1). He came round to the authors’ view that ‘the opening … should put the reader properly in the picture as to what it is all about’. Edwards had had a go by starting with the publicity—‘The world seemed to enjoy the arrival of Louise Brown almost as much as we did’—but the draft did not explain enough for Harris.^67^

Abse began again by going into full autobiographical mode—his own, that is. He worked up a story of sex education at a Cardiff primary school, then somewhat depersonalised this as he cut down his own drafts: ‘Probably eleven year old boys no longer sit shuffling in the chalk-smelling classrooms of Britain while a visiting tall stranger … talks to them about sex.’^68^ By the time he had finished, there remained for the opening of ‘The Quest’ only ‘Small children are sometimes told by their parents that babies are born because their mothers and fathers “love each other very much.” That leaves some of them puzzled … what of those childless aunts who have been married for many years?’ Eggs, sperm and infertility led to Steptoe’s student days at St George’s Hospital and his encountering women ‘who asked poignantly, “Why, doctor, can’t I have a baby?”’ Edwards commented, ‘Patrick needs much more sympathy and enthusiasm. “Aunts”—don’t like.’^69^ Steptoe told Abse, ‘I am enjoying the book enormously, but I thought the opening a little halting.’ Proud of his musical accomplishments, he offered a fulsome account of his own performance of a piano concerto for colleagues.^70^

Instead, Abse opened with the case of a woman suffering from infertility that he pulled from a hat for the student Steptoe to encounter. This established her distress and Steptoe’s compassion. The teaching context allowed the consultant to explain infertility and that blocked tubes stopped the egg from meeting the sperm. ‘The patient then asked an astonishing question’ and a convenient one: ‘“Doctor, can’t the Fallopian tubes which you say are blocked be by-passed?” “Oh no”, Mr Gwillim said’. Abse introduced in vitro fertilisation as a remote possibility being tried then. Quick paragraphs touched on the opposition Steptoe and Edwards faced, ethical considerations, scientific speculations, technical problems and setbacks before the quest culminated in ‘the longed for, normal cry of a … baby’. ‘[D]esperate’ women had been given ‘hope’.^71^ Here was the arc of the book.

## Marriages of the Minds

The quest for a baby contained Edwards’s and Steptoe’s searches for each other. That ‘romance of IVF’ was always going to structure the book, but Abse’s work with the authors distributed agency to their wives and made more space in the partnership for Edwards’s assistant Jean Purdy.^72^ This was, in the first place, not feminism but a consequence of Abse’s fleshing out supporting characters, including some men, and having ideas come up in dialogue. The interviews then gave Edwards opportunities to acknowledge more help.

From the start, the key scenes of the romance were Edwards’s discovering Steptoe’s laparoscopy in a Cambridge library in 1967, a phone call on which Edwards did not follow up and their face-to-face encounter at a conference months later. Abse enhanced the drama and dovetailing.^73^ Edwards had ended a chapter on his find and dedicated the next to the ‘Meeting of the Minds’. Abse elevated the discovery of the collaborator by focusing a chapter he called ‘Eureka’ on the passage from the library to the meeting. He then transitioned to Steptoe’s early chapters, which worked up to presenting the call and conference from his point of view.

The drafts are even more suggestive than the rewrite about the significance of the moment when Steptoe and Edwards met in person and both found ‘the man for me’. For Steptoe, ‘Although we have been backed up by wonderful teams of helpers nevertheless the economic size of the team—just two—has enabled us to understand each other[’]s problems quickly, and to come to rapid decisions. This marriage between the scientific and clinical approaches has been … long lasting and permanent … with no hint of divorce’. For Edwards, ‘Five minutes conversation and we were … eager to collaborate, obviously sharing many characteristics’ and ‘complementary in so many ways’. ‘Two’s company’, he wrote, and so implied ‘three’s a crowd’.^74^

Edwards and Steptoe starred in their own autobiography, of course, but revision made clearer that a team stood behind each of them. Collaboration began with Edwards’s love affair with fellow PhD student Ruth Fowler. Abse represented her, a scientist in the same field, as following the work and participating in some of it, active in decisions and proposing moves. But she still became the supportive wife, bringing up five children through Edwards’s frequent absences. Abse added a story that she, having suggested Edwards go to Baltimore in search of human eggs, then pretended when he phoned that all was fine, although in fact the whole family was down with the flu. That revelation started a chapter, even after Fowler protested, ‘NOT TRUE!’^75^ Sheena Steptoe née Kennedy, a former actor who no longer worked outside the home, had taken a less direct part. Abse made her an interlocutor, though without great lines (‘“Who’s he?” … “Off you go then”’).^76^

Steptoe had already praised his Oldham team, and he and Edwards dedicated the book first to ‘our loyal staff’. The pair particularly valued loyalty because they were embattled. Much space went on score-settling with molecular biologists, the gynaecological hierarchy, the MRC and the press. But since wrestling the establishment and vanquishing villains make a good story, Abse did not change much, and nor did Hutchinson’s lawyers.^77^ The key issue was the extent to which the stars would let someone else into their professional marriage.

**Figure 1.**
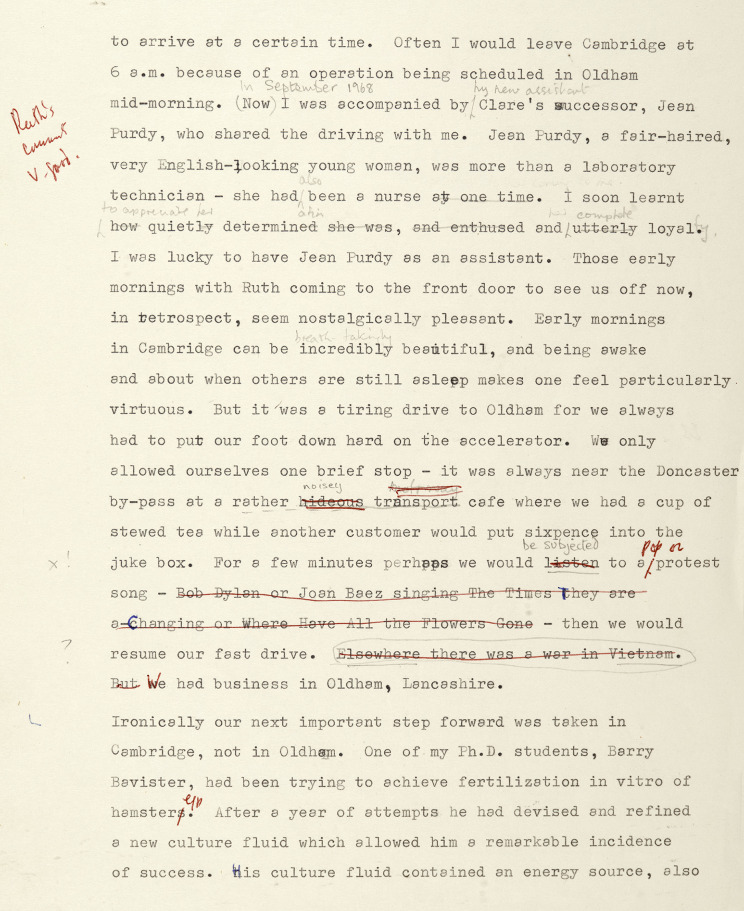
Page of ‘Bob’s corrections’. Robert Edwards’s annotations are in red pen, including ‘Ruth’s comments v. good’, Ruth Edwards’s in pencil towards the top, modifying the description of Jean Purdy, which was altered a little for publication. Purdy’s are in pencil below, conveying her dislike of popular music, while Edwards struck out what he perhaps saw as extraneous, but that passage was published unchanged. Dannie Abse Papers, folder 280, 96. National Library of Wales Collection.

Abse’s revisions turned Purdy into one of the cast. Edwards’s draft stated that she, like Clare Jackson, whom she replaced in 1968, had provided ‘ungrudging help’, itself a somewhat grudging acknowledgment. But Purdy evidently shared the work, the travelling and the waiting. Steptoe called her ‘more than a technician. As a former trained nurse, she has been fully involved in the motivation, clinical and scientific problems.’^78^ The revision made her a character through descriptions and dialogue, with more effusive thanks from Edwards to this ‘fair-haired, very English-looking young woman’. ‘I soon learnt how quietly determined she was, and enthused and utterly loyal’ (Fig. 1). Now, while still presenting himself as the innovator, he spelled out that ‘Jean’s cooperation had become crucial. It was no longer just Patrick and I. We had become a threesome.’^79^ On the one hand, even as revised *A Matter of Life* reinforced the view that the men made a team of two and women helped. On the other, Abse’s rewriting paved the way for Edwards’s campaigning for Purdy as deserving equal billing with himself and Steptoe and thus for recent celebrations of her contribution.^80^

Edwards often wrote ‘Jean and I’ because they shared tasks, but some labour was gendered. Abse had Steptoe assign credit for Louise Brown’s birth: ‘[Bob’s] brain, skill and perseverance … had led to this wonderful moment of achievement, and Jean’s hard-working devotion’. Edwards now recognised that, ‘As an ex-nurse and as a woman she was clearly able to identify herself with [the patients] in a special way. She would sometimes point out small but important things that Patrick and I, as mere men, had overlooked.’^81^ ‘Mere’, but by implication in charge of the big things.

Baby Louise was the fruit of a professional marriage which, as Abse shaped it, made room for the researchers’ wives, for Purdy, for members of the larger teams and for Lesley and John Brown, but this was still the doctors’ tale.

## Embryos and Patients

The women most intimately entangled in the project were the volunteers to whom Edwards and Steptoe had expressed gratitude for their willingness to have procedures tested on them, in part to help others.^82^ Abse fleshed out Steptoe’s early patients: a nursing sister with no alternative but laparotomy for what turned out not to be a dangerous condition; and another nurse, on whom, thanks to physician John Hirst, now also characterised as a tall, plain-speaking Yorkshireman, Steptoe performed his first successful laparoscopy on a living patient.^83^ Above all, Abse managed better the transition from embryos to volunteers.^84^

Edwards had described his epiphanies as seeing eggs and embryos under the microscope. Harris found these passages so ‘lyrical’ that he planned an embryo photo for the book jacket.^85^ Edwards wrote of the last such breakthrough,

Arriving in Oldham, I went straight to our small laboratory next to the operating theatre, to that tiny room, six foot by nine foot. Jean was waiting and she placed the embryos under the microscope for me to see. It was an unbelievable sight: four beautiful human blastocysts … the … disc of foetal cells at the beginning of their journey towards life. Light, transparent, floating, expanding slightly … there they were, four excellent blastocysts. I knew in an instant that we had reached our goal ….

Edwards had contextualised that achievement—which Purdy had seen and recognised first—in relation to fighting the temptation ‘to replace the blastocysts into the mother on the spot’. This was strong because ‘they belonged to the wife and husband who had given their eggs and spermatozoa, and not to us. We had merely made fertilisation possible’.^86^

In focusing the chapter on ‘Four beautiful human blastocysts’, it was probably Abse who deepened the performance of humility and changed its meaning as he expanded Edwards’s reflections into a meditation on ‘the wonders of Nature as they unfold in all their beauty. We had merely observed [the blastocysts] …; someone else designed them.’^87^ Now, ‘We had merely’ invoked a contrast not to parents but to a designer. It also took a few attempts to end the chapter well. They had arrived at, ‘As I walked to the car I looked up at all the stars, the moon, the night sky …’, when Purdy, who perhaps inflected awe through her Christianity, drove home the comparison by concluding, ‘and considered the equally amazing sights I had just seen under my microscope’.^88^ Embryos appeared as natural wonders in a cosmic drama.

Once the team started implanting, the embryos mattered for their ability to relieve patients’ suffering. Although it began clinically, Steptoe’s draft chapter ‘The Pregnancy’ detailed his meeting with Lesley and John Brown, describing them warmly and making clear the strain infertility had placed on their marriage. Abse added dialogue: ‘“I would be a good mother”, Mrs Brown said softly. It was quiet in my consulting room before John Brown said feelingly, “You see, doctor, she always wanted a child.”’ The new title, ‘A Patient Called Lesley Brown’, shifted the emphasis.^89^ The problem was how she had already been introduced.

In the previous draft chapter, ‘The Breakthrough’, Edwards told how he and Purdy began working with patients’ natural cycles rather than inducing superovulation. With that as the theme, Lesley Brown made her underwhelming debut as one of ‘our other two patients’ and was named only after the embryo transfer. Her presentation was fragmented further because the recovery of the egg that had developed into the embryo came after this. Abse fixed that, but it took a late reordering to bring Steptoe’s chapter forward, thereby presenting Brown as a patient and a person before she became the subject of procedures (Table 1).^90^

The transfer of the embryo into Brown is the second of two events—the first is their meeting in London—that, in the book, both Edwards and Steptoe still narrate. In Steptoe’s version, ‘Together Bob and I advanced the precious load into the body of the womb.’^91^ For Edwards, Abse included a comment (elicited at interview) by a ‘totally relaxed’ Mrs Brown: ‘“Cor”, she said to Jean, “that was a marvellous experience”’. This had ‘stuck in Jean’s mind’ because it seemed a ‘strange thing to say’.^92^ For Squier, writing about *A Matter of Life*, Edwards and Steptoe’s ‘accomplishment of IVF satisfied the unconscious fantasy of a joint male pregnancy’.^93^ The reality, complete with sexual overtones, was a joint impregnation.

Purdy accepted that passage but baulked at Steptoe’s description of preparing Brown for the Caesarean: ‘This whole page sounds awful—you loose [*sic*] sight of [L]esley under all the equipment—Is all this detail *really* necessary?’^94^ Already worked over by Harris, the text did not change. The Browns had related their experiences in a more fully ghostwritten autobiography, for which Harris warned Edwards not to give interviews.^95^ The star patient just needed to be set up so readers could appreciate the doctors’ achievement in producing the baby who, in the end—did the marketing department overrule Harris?—looked out from front and back covers. Eggs and embryos were relegated to two out of eight pages of black-and-white plates and a few diagrams. Lesley Brown and Grace Montgomery, the second ‘happy mother’ from the Oldham programme, were pictured ‘with babies which they had been told they could never have’.^96^

**Figure 2.**
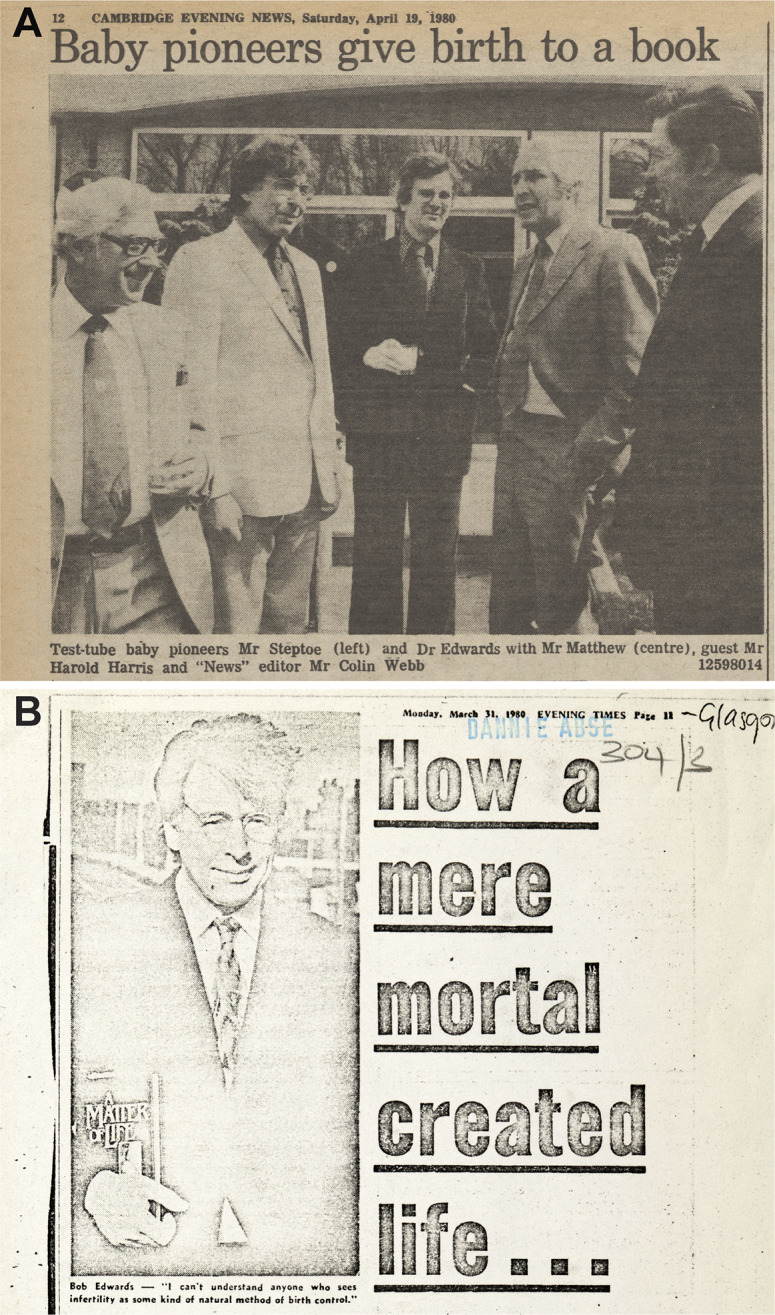
The authors publicise *A Matter of Life.* *A*, Photo of Steptoe, Edwards, Christopher Matthew (author, *Diary of a Somebody*), Harold Harris and *Cambridge Evening News* editor Colin Webb on the occasion of a ‘literary lunch’ at the Garden House Hotel. Publicity claimed: ‘This book has been described as the most important work of popular scientific interest since “The Double Helix.”’ From ‘Baby Pioneers Give Birth to a Book’, *Cambridge Evening News*, 19 April 1980, 12, by permission of Reach Publishing Services Ltd and the Syndics of Cambridge University Library. *B*, Portrait photo of Edwards with book as accoutrement next to a hubristic headline in Jessica Barrett, ‘How a Mere Mortal Created Life’, *Evening Times* (Glasgow), 31 March 1980, 11. No original could be found, so reproduction is from a photocopy in the Dannie Abse Papers, folder 304/3, by permission of Newsquest Media Group and the National Library of Wales.

The emotion peaked in the scene when Steptoe handed Lesley Brown her baby. As revised, this culminated in the sentence ‘I doubt I shall ever share such a moment in my life again.’^97^ Abse had managed things so that readers would realise the significance. Then, like a surrogate mother, he moved into the background. He wrote most of what readers have found most striking, but was absent when a newspaper announced, ‘Baby pioneers give birth to a book’, and from celebrations that featured Edwards, Steptoe and occasionally Harris (Fig. 2A).^98^

## ‘Their Own Moving Story’

**Figure 3.**
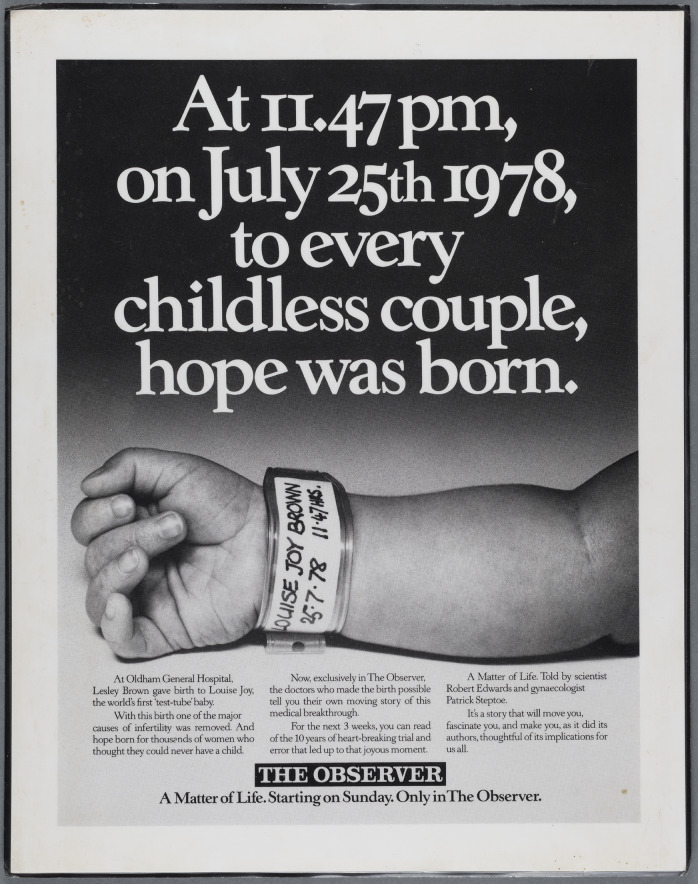
Ad for the serialisation of *A Matter of Life* in *The Observer.* It ran in newspapers (including *The Guardian*, 15 March 1980, 3, and *Daily Mail*, 15 March 1980, 8), but this photo is of a laminated A4 poster in Lesley Brown’s Papers, Bristol Archives, 45827/PU/4. © Guardian News & Media Ltd 2025.

Harris had proposed the title ‘Life in Vitro’. ‘Even if nobody understands it, they soon will’, he confided optimistically. ‘There is a hint of mystery about it and of drama. After all, nobody knew what a helix was until *The Double Helix.*’ Abse suggested ‘Out of the Test Tube’, which Harris expected the authors to reject because they disliked the term ‘test-tube baby’.^99^ As the blander *A Matter of Life*, the book was published in the United Kingdom at the end of March 1980, just before Edwards and Steptoe’s clinic opened and others began to replicate their success. A round of publicity promoted the definitive account of the making of IVF. The ad for the serialisation over the previous two weeks had announced: ‘At 11.47 pm, on July 25th 1978, to every childless couple, hope was born. … Now, exclusively in *The Observer*, the doctors … tell you their own moving story of this medical breakthrough’ (Fig. 3). It was their story but Abse’s rewrite gave it the power to move readers.

Most reviewers hailed ‘an enthralling story of dogged endeavour by two patient, hardworking men’.^100^ Quita Morgan in the Bristol *Evening Post* ‘literally could not put [it] down. Except once—and, oh the embarrassment of this admission for a supposedly hardbitten journalist: my eyes refused to read on at one stage because tears got in the way.’ For the *Birmingham Post*, the chapters ‘dovetail effortlessly … to make a thriller as exciting as anything an author of fiction could dream up. But their story also transposes into solid flesh and blood the somewhat shadowy and sometimes almost sinister figures that have lurked behind the headlines, so that we meet them at last as they really are, compassionate and at times very vulnerable human beings, utterly dedicated to the task they set themselves—to help infertile women’. Edwards told Jean Smith of *The Scotsman* ‘that perhaps he hasn’t said enough about … other [infertile] patients who co-operated. “Perhaps I’ve dwelt too much on the opposition ….” … Some of his colleagues, he feels, may be critical of the way in which he has tried to make the book readable. And readable it is.’ Edwards thus claimed Abse’s work and was pictured with the book (Fig. 2B).^101^

A few critics complained that *A Matter of Life* was ‘atrociously ill-written’ and fewer commented on Abse’s role. Margaret Forster, author of *Georgy Girl*, wrote in the London *Evening Standard* that ‘Dannie Abse, to whom the authors pay tribute for his help, seems to have saved them from few amateurish blunders’. She gave an example that, late in the day, he did apparently write for Chapter 1: ‘“I thought about her plight”, Steptoe writes, “as I played the piano at my digs, played perhaps a Beethoven or Schubert sonata, as was my wont”’. Yet in the end she, too, was ‘on my feet applauding’.^102^ For the *Sunday Times*, smarting from being beaten to the serialisation, science correspondent Bryan Silcock argued that ‘the sheer grind … comes across clearly enough. But neither author shows any of the novelist’s gifts which made … *The Double Helix* such a riveting read …. Edwards and Steptoe plod along their chosen road’. Though in the end, ‘it is impossible not to become involved’.^103^

The *British Medical Journal* placed the book in the debates about medicine and the media that had loomed so large in the coverage of the birth. The TV producer and writer Karl Sabbagh lampooned the style:

I opened the slim, black-covered volume on the 1815 to Cardiff. … It was raining outside, and … people went about their business, unaware that I was about to start reviewing *A Matter of Life* …. Elsewhere there were Russian troops in Afghanistan, but I had business in Cardiff, Wales. … I settled down to read … the story of two persistent men who against all odds brought happiness to women whose tears threatened to wash their marriages away. Steptoe and Edwards; such a curious combination of names. … How had these two come together? The patrician consultant venerable in years and the young Yorkshire lad who got only an ordinary degree at Bangor and began to wonder about Life with a capital L. …

I’m sorry. I can’t keep it up for more than a couple of hundred words, and I’m surprised that Steptoe and Edwards managed so valiantly to write a whole book in this women’s magazine style. Or did they? If they wrote it unaided they appear not to have read it very carefully after it was finished.

Sabbagh took exception to repetitions, especially those that varied Lesley Brown’s words. His destination may veil a dig at Abse. Thematising travel critiqued the emphasis on driving between Cambridge and Oldham, which dominated Edwards’s life (and Purdy’s) in the 1970s but Sabbagh found ‘humdrum’. ‘Elsewhere’, a favourite Abse link, recalled his shoehorning in of the Vietnam War.^104^

Sabbagh hated that ‘Steptoe and Edwards have fallen into the hands of the media they criticise so heavily in the book’.^105^ Recalling Crick’s comment to Watson—‘Your view of history is that found in the lower class of women’s magazines’—the gender snobbery tarred a book by association with a genre seen as prioritising ‘gossip’ over ‘intellectual content’.^106^ But suiting a transgressive technology for safe periodicals might have counted as a success.

Almost every reviewer approved of IVF, but in the Newcastle *Journal* Sue Hercombe, still ‘dismayed by the suggestion that procreation can properly be separated from an act of sexual love’, made a telling point: the ‘sentimental identification with the feelings of couples who could not produce offspring’ struck ‘a false note in Mr Edwards’ unromantic world of cultures, spermatozoa, ovarian tissue and fallopian tubes’.^107^ In time, radical feminists would cite *A Matter of Life* as confirming that men did IVF to women.^108^ Despite Abse’s additions, it could be read as describing the ‘fortuitous dovetailing’ of ‘a research technique’ and ‘infertility treatment’.^109^

Meanwhile the authors, reckoning that the more people understood, the more would accept their work, did publicity in Britain and Steptoe gave interviews in Canada and the United States (Fig. 2).^110^ Morrow, the US publisher, advertised the tour, a cover price of $9.95, a first printing of 35,000, a ‘$30,000 ad/promo budget’ and status as a Book of the Month Club alternate selection.^111^ A reviewer opined during that visit, ‘We are not yet used to the idea of doctors as superstars … but in acting the part in the promotion of this book Edwards and Steptoe have … done a lot to prompt open discussion of reproductive technology and a public awareness of … ethical dilemmas.’^112^ With paperbacks and a global reception, including other serialisations and a few translations, the autobiography became the standard account for supporters and opponents who unknowingly copied and commented on Abse’s prose.^113^ Paradoxically, the scenes he constructed include the most vivid and seemingly direct. The book has thus been hard to challenge, though a few eventually did, to an extent.

When Edwards won the Nobel Prize in 2010 (Steptoe, like Purdy, had died in the 1980s), he was incapacitated by illness but Ruth Edwards pushed for a second edition. She insisted on revision, though, and not just to bring the text into line with twenty-first-century ‘sensitivities’ about animal rights and disability and to include ‘IVF’ on the cover. Far more changes undid those parts of Abse’s work to which she and her husband had objected in 1979. For Random House, which had taken over Hutchinson and had ‘[n]o desire to republish really’, the proposed revisions made a print-on-demand paperback and e-book ‘unworkable’. With Steptoe’s son Andrew, she took the book to Amazon instead. The revisions shed light on the families’ contrasting responses to the ghostwriting.^114^

The Edwardses’ daughter, Jenny Joy, told me as I was embarking on this research that ‘we as a family think *A Matter of Life* had so many mistakes in it that our Father could never have read it through properly’.^115^ The project, which had been ‘a distraction’ for him, though necessary ‘to get some of the story out’ (after criticism for not doing so), had come at ‘an extraordinarily busy time’.^116^ In revising, Ruth Edwards did not necessarily recall the corrections requested thirty years before, and is unlikely to have worked from any original manuscript, but reacted in similar ways. She excised ‘[b]iblical references’ that ‘do not sit easily with the sentiments of the rest of the text’.^117^ She cut ‘very, very flowery language that was not suitable for a more modern audience’ and the passages about ‘our Caroline, our Jennifer’ that ‘were not him *at all*’. She removed parts, such as the flu story and a reference to her ironing, which were untrue,^118^ and had presented her as a homemaker rather than a companion in science. By contrast, Patrick Steptoe had had more time for his chapters, which had been revised less, and ‘liked the end product’. This was a ‘more … popular’ book than he would have written and ‘some parts … don’t really sound much like him’, but Andrew Steptoe felt ‘that it should be their work as far as possible, as it were’, and edited only the little that struck him as ‘egregious’.^119^

Ultimately, the Amazon edition has enjoyed no great success and all of its changes, like the historical research led by Edwards’s students since 2008, have so far exorcised only small parts of Abse’s ghostly work. The screenwriters of *Joy*, connected to IVF as patients, took the ‘beautiful book’ by ‘Patrick and Bob’ as ‘our base’; ‘everything else we tried to build on top of that’. They then amplified Purdy’s role and those of the disappointed volunteers, but downplayed that of Ruth Edwards.^120^ Poetic licence included magicking away obstacles to participation, including by inserting Purdy into scenes where she was not present (Edwards’s first meeting with Steptoe) and that did not happen (an interview at the MRC), and speculating, contrary to the discussion of using natural cycles in *A Matter of Life*, that this was her idea.^121^ But taking liberties and shifting contributions was nothing new. The process began, we can now see, when Abse rewrote the authors’ drafts to make a no less commercial product. For better and worse—and it is both—he still haunts the history of IVF.

## Conclusion

Biography and autobiography, the major genres of mass-market science communication, owe much of their appeal to collaboration. Certainly, the book that has most shaped histories of assisted reproduction cannot be grasped without considering the ghostwriter’s work to develop structures and tropes. Abse exaggerated the drive to alleviate infertility so much that it jarred with Edwards’s primary enthusiasm for mammalian genetics and embryology, though it gave coherence to the joint story and aided his advocacy for reproductive biomedicine. Abse enlarged the cast of named, active characters and so helped Edwards and Steptoe go some way towards recognising others’ contributions. He gave their wives more to do, had Edwards acknowledge Purdy more and ensured that Lesley Brown appeared first as a whole patient. The gender politics remain in many ways unreconstructed, and mere inclusion has its limits in representing the inputs of marginalised actors, but a feminist ghost would have faced an impossible task. In that respect, the market has changed.

Hutchinson’s business imperative, Edwards and Steptoe’s desire to promote IVF, acceptance that they needed help and the former’s lack of time gave Abse power. He ventriloquised but did not disappear into either author’s identity as he juggled two contrasting characters and added colour and literary culture, especially to Edwards’s draft. The harvest from the interviews mostly let him and Steptoe come across better—more engaging, more generous, more committed to helping patients—as Harris had promised. But the ‘burying’ of their lives is blatant in the changes of voice and where ghost and publisher rebuffed corrections. Critics also had a point that *A Matter of Life* is uneven, despite the talent involved. A gig to subsidise Abse’s poetry and his own prose did not demand perfection, and the publisher and most reviewers were content. The principals claimed the book as their own and, if they took flak for its being so commercial, they had suffered worse.

Among the minority of scientists and physicians who have authored book-length lives, most had less help than Edwards and Steptoe. Where subjects understood themselves as good with words, hiring a writer seemed superfluous and risked a loss of status as well as authenticity. After advice from family, friends, referees and editors, a few memoirs were hailed for their literary merit. More could have used a rewrite, but for a long time only the most celebrated had that option. Today, medics write many autobiographical books, but leading innovators are not expected to have the leisure for immersion in literary culture. They may thrive in other collaborative media, notably radio interviews.^122^ But those who choose to author autobiographies increasingly employ writers to help tell their tales. If the genesis of *A Matter of Life* is any guide, these ghosts—now often credited as co-authors—fit the raw materials to generic conventions as they add and subtract. Whether they write the first draft or the last, such collaborations sculpt stories of discovery and invention.

In other words, scientific and medical autobiographies are, with respect to ghostwriting, like others. Their analysis can thus contribute to interpreting collaborative autobiography more generally. The records available for this one make clearer than usual how a book doctor’s work with the authors shaped the cast. Yet biographical presentations of science and medicine also rely on particular sets of expectations. Features such as the consistent quest, the struggle to relieve suffering, the campaign for control of nature and triumph over benighted opposition are so ingrained that they may not need a ghost to bring them in. Nor are first drafts necessarily more accurate or personal, though research should consider them. But when ghostwriting fits a book to a market, it tends to tighten the constraints of custom and so to pull the narrative in characteristic ways. Fresh audiences may then facilitate new collaborations. To make the results not just more empowering but also truer than the prevailing accounts, it helps to appreciate how these were produced.

## Footnotes

1

Michael Shortland and Richard Yeo (eds), Telling Lives in Science: Essays on Scientific Biography (Cambridge: Cambridge University Press, 1996); Donald Pollock , ‘Physician Autobiography: Narrative and the Social History of Medicine’, in Narrative and the Cultural Construction of Illness and Healing, ed. Cheryl Mattingly and Linda C. Garro (Berkeley: University of California Press, 2000), 108–27; Lesley Graham , ‘Scientific Autobiography: Some Characteristics of the Genre’, ASp, 43–4 (2004), 57–67 .

2 Ben Taylor (dir.), *Joy* (Netflix, 2024); Martin H. Johnson and Kay Elder , ‘The Oldham Notebooks: An Analysis of the Development of IVF, 1969–1978. V. The Role of Jean Purdy Reassessed’, Reproductive BioMedicine & Society Online, 1 (2015), 46–57 28299364 10.1016/j.rbms.2015.04.005PMC5341260; Roger Gosden , ‘Jean Marian Purdy Remembered: The Hidden Life of an IVF Pioneer’, Human Fertility, 21 (2018), 86–928881151 10.1080/14647273.2017.1351042.

3 Jack Thorne, ‘“Rachel Had Been Ready to Leave Me if Our IVF Hadn’t Worked”’, *The Guardian*, 10 November 2024, https://www.theguardian.com/film/2024/nov/10/screenwriter-jack-thorne-ivf-joy-film-netflix; Robert Edwards and Patrick Steptoe, *A Matter of Life: The Story of a Medical Breakthrough* (London: Hutchinson, 1980) (hereafter ***AMoL***).

4

Susan Merrill Squier , Babies in Bottles: Twentieth-Century Visions of Reproductive Technology (New Brunswick, NJ: Rutgers University Press, 1994), 158–65. For excerpts: Robin Morantz Henig , Pandora’s Baby: How the First Test Tube Babies Sparked the Reproductive Revolution (Boston: Houghton Mifflin, 2004), 32–3, 49, 51, 61, 74, 76, 79, 85, 170–2, 202; Philip Ball , Unnatural: The Heretical Idea of Making People (London: Vintage, 2012), 196–7, 217–19; for the research: Martin H. Johnson et al., ‘Why the Medical Research Council Refused Robert Edwards and Patrick Steptoe Support for Research on Human Conception in 1971’, Human Reproduction, 25 (2010), 2157–7420657027 10.1093/humrep/deq155PMC2922998; Johnson , ‘Robert Edwards: The Path to IVF’, Reproductive BioMedicine Online, 23 (2011), 245–6221680248 10.1016/j.rbmo.2011.04.010PMC3171154; Johnson and Kay Elder , ‘Symposium: The History of the First IVF Births’, Reproductive Biomedicine & Society Online, 1 (1) (2015), 3–70 28299360 10.1016/j.rbms.2015.04.001PMC5341285; Roger Gosden , Let There Be Life: An Intimate Portrait of Robert Edwards and His IVF Revolution (Williamsburg, VA: Jamestown Bookworks, 2019); Nick Hopwood , ‘Reconstructing Robert Edwards: Biography and the History of Reproduction’, Reproductive BioMedicine Online, 40 (2020), 605–1232280012 10.1016/j.rbmo.2020.02.010. Steptoe has been less studied, in part for lack of acolytes or archives, but see R.G. Edwards , ‘Patrick Christopher Steptoe, CBE, 9 June 1913 – 22 March 1988’, Biographical Memoirs of Fellows of the Royal Society, 42 (1996), 435–5211619339.

5

Philippe Lejeune , On Autobiography, ed. Paul John Eakin , transl. Katherine Leary (Minneapolis: University of Minnesota Press, 1988), 188 .

6 Squier, *op. cit.* (n. 4), 12.

7

Eva Shaw , Ghostwriting: How to Get into the Business (New York: Paragon House, 1991); Andrew Crofts , Ghostwriting (London: Black, 2004); Jennie Erdal , Ghosting: A Memoir (Edinburgh: Canongate, 2004); Robert Harris , The Ghost (London: Hutchinson, 2007); Hernan Diaz , Trust (London: Picador, 2022).

8

David Finkelstein ‘Unraveling Speke: The Unknown Revision of an African Exploration Classic’, History in Africa, 30 (2003), 117–32, quotation on 121; further: André van der Velden , ‘Life Writing, Marketing and the Construction of Cinema History: On the Ghostwritten Autobiography of Dutch Film Entrepreneur Abraham Tuschinski’, in Arianne Baggerman , Rudolf Dekker and Michael Mascuch (eds), Controlling Time and Shaping the Self: Developments in Autobiographical Writing since the Sixteenth Century (Leiden: Brill, 2011), 331–53 ; Andrew Mumford and Katherine Bayford , ‘Ghostwriting History: Churchill, Kennedy and the Authenticity of Authorship’, International History Review, 47 (2025), 54–69 .

9 Especially in the controversy over Manning Marable , Malcolm X: A Life of Reinvention (London: Allen Lane, 2011).

10 Dannie Abse Papers, National Library of Wales Collection (hereafter **DAP**), ‘Ghostwriting’ folders, 277–308. The interviews are represented in questions and notes not recordings or transcripts. I lack direct evidence of copy-editing or proof-reading, but the text changed little between Abse’s last extant manuscript and the book.

11 This has hardly been thematised although it is known, for example, that *‘Surely You’re Joking, Mr. Feynman!’*, the most successful autobiographical writing by a physicist, was edited from a transcript of conversations with a friend: James Gleick , Genius: The Life and Science of Richard Feynman (New York: Pantheon, 1992), 408–11.

12

G. Thomas Couser , Vulnerable Subjects: Ethics and Life Writing (Ithaca, NY: Cornell University Press, 2004), ch. 3.

13 Harris, *op cit.* (n. 7), 6.

14

James D. Watson , The Annotated and Illustrated Double Helix, ed. Alexander Gann and Jan Witkowski (New York: Simon & Schuster, 2012).

15 Squier, *op. cit.* (n. 4), 165.

16 Examples: Kary Mullis , Dancing Naked in the Mind Field (New York: Pantheon, 1998), of which David Fisher wrote the first draft; Ian Wilmut , Keith Campbell and Colin Tudge , The Second Creation: Dolly and the Age of Biological Control (Cambridge, MA: Harvard University Press, 2000); Edward Teller with Judith L. Shoolery , Memoirs: A Twentieth-Century Journey in Science and Politics (Cambridge, MA: Perseus Publishing, 2001); Magdalena Zernicka-Goetz and Roger Highfield , The Dance of Life: The New Science of How a Single Cell Becomes a Human Being (London: Allen, 2020); Katalin Karikó , Breaking Through: My Life in Science (New York: Crown, 2023), on which see Ali Benjamin, https://www.alibenjamin.com/cowritten-books, last accessed 7 December 2024.

17 Edward Candy [pseud. Alison Neville], ‘We Are Not Entitled’, *The Times*, 11 April 1970, ‘Saturday Review’, v; Malcolm Muggeridge, ‘Not a Dog but a Man’, *Observer Review*, 29 March 1970, 25; Marjorie Proops, ‘My Heart and I’, *Daily Mirror*, 25 March 1970, 15; on Barnard’s British reception: Ayesha Nathoo , Hearts Exposed: Transplants and the Media in 1960s Britain (Basingstoke: Palgrave Macmillan, 2009).

18 Harry Pugh and Frank Welsby, ‘Baby of the Century’, *Daily Express*, 11 July 1978, 1; works cited in note 4.

19

Katharine Dow , ‘“The Men Who Made the Breakthrough”: How the British Press Represented Patrick Steptoe and Robert Edwards in 1978’, Reproductive BioMedicine & Society Online, 4 (2017), 59–67 29774267 10.1016/j.rbms.2017.07.002PMC5952836; Dow, ‘Looking into the Test Tube: The Birth of IVF on British Television’, *Medical History*, 63 (2019), 189–208; Nick Hopwood , ‘Artificial Fertilization’ and ‘“It’s a Girl”’, in Hopwood, Rebecca Flemming and Lauren Kassell (eds), Reproduction: Antiquity to the Present Day (Cambridge: Cambridge University Press, 2018), 581–96 and exhibit 38; Fiona Kisby Littleton , Susan Bewley and James Owen Drife , Presenting the First Test-Tube Baby: The Edwards and Steptoe Lecture of 1979 (Cambridge: Cambridge University Press, 2023).

20
*AMoL*, 167. In unused draft material (DAP 279/28, 21–2) Edwards wrote of offers ranging from ‘a quarter of a million pounds’ to ‘seven figures’, which became ‘very, very tempting’ when Steptoe was about to retire and they would have no clinic, but ‘We decided against signing contracts with anyone, because our story was too big for any one newspaper.’

21 Elliot Philipp, ‘Harold Harris’, *The Independent*, 12 August 1993, 24.

22 Harold Harris to Robert Edwards, 3 August 1978, Robert Edwards Papers, Churchill Archives Centre, Churchill College, Cambridge (hereafter **EDWS**), 4/14/2.

23 Contracts signed 5 September 1978, ‘*A Matter of Life*: Steptoe, Edwards, Abse etc. contracts & paperwork’, Hutchinson Archive, Penguin Random House Archive & Library, Rushden, Northamptonshire (hereafter **PRHA**).

24 ‘Book on the first test-tube baby’, [12 September 1978], EDWS 4/14/1.

25 Ion Trewin, ‘Winning the “Rights” Game at Frankfurt’, *The Times*, 21 October 1978, 12.

26 Harris to Edwards, 11 September 1978 (setting 28 February 1979 as delivery date), 12 and 21 February and copy of Edwards to Harris, 27 February 1979, EDWS 4/14/1; Patrick Steptoe to Harris, 6 March 1979, DAP 277; Steptoe to Harris, 9 January; Diana Mann to Harris, 14 and 28 February; Edwards to Harris, 23 February 1979, DAP 307/1–3, /5.

27 ‘Book’, *op. cit.* (n. 24).

28 DAP 277, ‘Contents’.

29 Harris to Steptoe and Edwards, 28 November 1978, EDWS 4/14/2; copy of Edwards to Harris, 27 February 1979, EDWS 4/14/1.

30 Copy of Harris to Edwards, 26 February 1979, DAP 307/4/1.

31 Copy of Harris to Steptoe and Edwards, 9 March 1979, DAP 307/6/1.

32 Harris to Steptoe and Edwards, 28 November 1978, EDWS 4/14/2. The column was ‘All in a Doctor’s Day’ by Cedric Carne: Keren Abse to author, 17 March 2025. Dannie Abse’s account of the lunch is consistent with late November, but he appears to have brought forward his impressions of the manuscript: Dannie Abse , Goodbye, Twentieth Century: An Autobiography (London: Pimlico, 2001), 239–41.

33 Harris to Edwards, 21 February 1979, EDWS 14/4/1.

34
*Ibid*.; Abse, *op. cit.* (n. 32), 240–3, though Edwards had drafted most of what Abse here presented as his: DAP 277, ‘Chapter Eight: The Good Years’, 11, 12, 15. For the addendum bringing Abse into the agreement (but not assigning him copyright): EDWS 4/14/2; ‘*A Matter of Life*: Steptoe, Edwards, Abse etc. contracts & paperwork’, PRHA, where on one copy he is noted as ‘rewriter’.

35 Harris to Edwards, 17 August 1979, EDWS 4/14/1.

36 Dannie Abse, *Ash on a Young Man’s Sleeve*, 2nd edn (Oxford: Pergamon, 1969); Abse, *A Poet in the Family* (London: Hutchinson, 1974). Harris lent Edwards the latter: Harris to Edwards, 26 February 1979, DAP 307/4/3. On Abse: Tony Curtis, *Dannie Abse* (Cardiff: University of Wales Press, 1985); Curtis, ‘“We Keep the Bread and Wine for Show”: Consistent Irony and Reluctant Faith in the Poetry of Dannie Abse’, *Proceedings of the British Academy*, 154 (2008), 337–60; W. Richard Bowen, ‘Nourished by Experiences: Meaning without Metaphysics in the Poetry of Dannie Abse’, *International Journal of Welsh Writing in English*, 6 (1) (2019), https://ijwwe.uwp.co.uk/article/id/579/.

37

Dannie Abse , A Strong Dose of Myself (London: Hutchinson, 1983), 198–200 .

38 Abse, ‘White Coat, Purple Coat’, quoted in Curtis, ‘“We Keep”’, *op. cit.* (n. 36), 338.

39 Abse, *Ash*, *op. cit.* (n. 36), 43, also 150–1; Curtis, *Dannie Abse*, *op. cit.* (n. 36), 12–15.

40 Abse, *op. cit.* (n. 32), 241.

41
*Ibid.*, 240.

42 DAP 277, ‘Chapter Two: Bob’, 1, 3. Edwards wrote that ‘the agriculturists … barred their Honours Course to me and I had to leave the subject with a deep sense of failure’, but Abse made the switch his decision. On such rescues: Pnina G. Abir-Am , ‘Nobelesse Oblige: Lives of Molecular Biologists’, Isis, 82 (1991), 326–43, on 3301917436 10.1086/355756.

43 DAP 278/11–14.

44 DAP 277, [Questions for Edwards]; 278/14, 1.

45 DAP 277, ‘Chapter Two: Bob’, 3–4; 278/14, 2.

46 DAP 280, 7; *AMoL*, 16.

47 DAP 278/1, /11–14.

48 DAP 277, ‘Chapter One’, 3.

49 DAP 280, 7.

50
*Ibid.*, 9–10.

51

Dannie Abse , Collected Poems, 1948–1976 (London: Hutchinson, 1977), xi (‘right word’ quoted); Abse, *op. cit.* (n. 37), 42 (‘I like’).

52 DAP 278/17–19, /21.

53 DAP 278/1–2, /15; 280, 19 (plays).

54 Rather than emphasise that working-class origins shaped a world view, Abse followed Edwards in introducing politics only once he joined the Labour Party around 1969 and was elected a city councillor in 1973: DAP 277, ‘Chapter Seven <9>: The Difficult Years’, 12; *AMoL*, 86. Edwards’s biographer has him as a lifelong socialist (Gosden, *op. cit.* [n. 4], 27), but in the late 1950s ‘<my admiration of> Jo Grimond … <made me join the> Liberal Party … <although I had always considered myself a labour supporter>’: DAP 280, 33; also 278/22, 36A.

55 DAP 280, 17–18.

56 Curtis, ‘“We Keep”’, *op. cit.* (n. 36), 358.

57 Squier, *op. cit.* (n. 4), 159.

58 Johnson, *op. cit.* (n. 4); Hopwood, *op. cit.* (n. 4), 607–8. Dannie Abse, *Medicine on Trial* (London: Aldus Books, 1967), 338, discussed Edwards’s work as a means of selection without mentioning infertility. In his draft, Edwards puzzled over what became a detour into immunology from 1957, when the potential for human embryology had perhaps dawned, to 1962, when success in maturing eggs ‘put me firmly and decisively into the study of human embryology’, or 1965, the ‘decisive turning point’, when he ‘was committed to the human work’. These were ‘the topsy-turvy years’ of ‘dodg[ing] backwards and forwards from eggs to immunology’ while searching for ‘medical partners’: DAP 277, ‘Chapter 3: Edinburgh: The Beginnings of an Idea’, 10; ‘Chapter Four: The Topsy-Turvy Years’, 7, 12, 15; ‘3: The Mouse House’, 12.

59 DAP 278/16, B (quotation), also /1.

60 DAP 280, 12, 14. Anne McLaren and John Biggers were not credited for births after embryo transfer in mice.

61 DAP 277, ‘Chapter Four: The Topsy-Turvy Years’, 3; *AMoL*, 38.

62 DAP 277, ‘Chapter Four: The Topsy-Turvy Years’, 3; 280, 37.

63 DAP 277, ‘Chapter Four: The Topsy-Turvy Years’, 7; 280, 46, 39.

64 DAP 277, ‘Chapter Four: The Topsy-Turvy Years’, 6; 280, 43–4, 48; for Abse’s valuing scripture: Bowen, *op. cit.* (n. 36).

65 DAP 280, 103; *AMoL*, 86.

66 DAP 277, ‘Steptoe: Early Years to End of War’, 21; 280, 70–5; *AMoL*, 63–6; further: Abse, *op. cit.* (n. 32), 240.

67 DAP 277, ‘Chapter One’, 1; copy of Harris to Steptoe and Edwards, 9 March 1979, DAP 307/6/1. DAP 277, ‘Introductory Comment’ implies a plan to use Edwards’s ‘Chapter 3’ and Steptoe’s ‘Final Chapter’ as samples, but the package sent with Gill Brogden to Edwards, 12 September 1978, EDWS 4/14/1, includes only Harris’s edit of Steptoe’s, which trimmed it and converted third person to first.

68 DAP 278/8, 1.

69 DAP 280, 1–2.

70 DAP 307/10.

71 DAP 282/6/2; further: Sarah Franklin, ‘Deconstructing “Desperateness”: The Social Construction of Infertility in Popular Representations of New Reproductive Technologies’, in Maureen McNeil, Ian Varcoe and Steven Yearley (eds), *The New Reproductive Technologies* (Basingstoke: Macmillan, 1990), 200–29.

72 For the romance: Squier, *op. cit.* (n. 4), 164; on Purdy: Johnson and Elder, *op. cit.* (n. 2); Gosden, *op. cit.* (n. 2); further: Helena M. Pycior, Nancy G. Slack and Pnina G. Abir-Am (eds), *Creative Couples in the Sciences* (New Brunswick, NJ: Rutgers University Press, 1996); Jenny Bangham, Xan Chacko and Judith Kaplan (eds), *Invisible Labour in Modern Science* (Lanham, MD: Rowman & Littlefield, 2022).

73 Edwards’s draft places the library find ‘some months’ before the call, but (inconsistently?) both in autumn, he having decided in the interim to ‘tak[e] the plunge’ and phone, while Steptoe had ‘heard about a scientist in Cambridge’ and ‘let it be known that maybe I could be of some help’: DAP 277, ‘Chapter Four: The Topsy-Turvy Years’, 20; ‘Chapter Seven: Meeting of the Minds’, 1–3; ‘Chap[ter] 6’, 4. This explains how Steptoe agreed so readily to collaborate. On the paper Edwards read: Johnson, *op. cit.* (n. 4), 254–6.

74 DAP 277, ‘Chap[ter] 6’, 6; ‘Chapter Seven: Meeting of the Minds’, 5–6.

75 DAP 280, 61. On Fowler: Simon Fishel , ‘Ruth Fowler (1930–2013)’, Reproductive BioMedicine Online, 28 (2014), 3–4 10.1016/j.rbmo.2013.11.01624444816.

76 DAP 280, 80.

77 Michael Rubinstein to Harris, 14 December; Harris to Edwards and Steptoe, 17 December 1979; copy of Edwards to Harris, 5 January 1980, EDWS 4/14/3.

78 DAP 277, ‘Chapter Seven: Meeting of the Minds’, 8; ‘Chap[ter] 6’, 7. Steptoe had credited Purdy for a ‘well designed piece of apparatus to be used at laparoscopy for the collection of eggs’: ‘Chap[ter] 6’, 8. The rewrite put this in a chapter by Edwards, but Purdy noted ‘wasn’t me sir’ and he changed it to ‘Jean and I’: DAP 280, 104.

79 DAP 280, 96, 149. ‘“Little Miss Reflected Glory”’ under Purdy’s name in a notebook strikes a jarring note; it is unclear whose view that was, but Steptoe earlier told Abse that matron Muriel Harris ‘felt Jean had privileges other nurses didn’t have’: DAP 278/3.

80 Johnson and Elder, *op. cit.* (n. 2); Gosden, *op. cit.* (n. 2); Martin H. Johnson, ‘IVF: The Women Who Helped Make It Happen’, *Reproductive BioMedicine & Society Online*, 8 (2019), 1–6.

81 DAP 280, 231, 149. Lesley and John Brown with Sue Freeman, *Our Miracle Called Louise: A Parents’ Story* (Wembley: Leisure Circle, 1979), was dedicated not just to Steptoe and Edwards (and Lesley’s Gran), but also to Sheena Steptoe and Jean Purdy, ‘who have been such kind friends’.

82 DAP 277, ‘Chapter Four: The Topsy-Turvy Years’, 19; ‘Steptoe: Early Years to End of War’, 23.

83 DAP 280, 77–8, 81–2.

84 Squier, *op. cit.* (n. 4), drew out the ‘deep cultural connection between reproductive technology and visualization technology’ (159) that *AMoL* sets up for Edwards’s microscopy and Steptoe’s laparoscopy, but since Abse did not consistently enhance this, I have not thematised it here.

85 Harris to Edwards, 31 July 1979, EDWS 4/14/1.

86 DAP 277, ‘Chapter Eight: The Good Years’, 15–16; on the blastocysts: Hopwood, *op. cit.* (n. 4), 610.

87
*AMoL*, 94–5. An insertion is marked in DAP 280, 114, but not included in the folder.

88 DAP 280, 114.

89 DAP 279/15–17; 280, 189–93.

90 DAP 277, ‘Chapter 11: The Breakthrough’, 5; 280, 172–9; *AMoL*, chs 18–21.

91 DAP 277, ‘Ch[apter] 12 continued’, 6; *AMoL*, 151.

92 DAP 278/3; 280, 177; compare 277, ‘Chapter 11: The Breakthrough’, 7.

93 Squier, *op. cit.* (n. 4), 165.

94 DAP 280, 226.

95 Harris to Edwards, 30 July 1979, EDWS 4/14/1; Brown and Brown, *op. cit.* (n. 81).

96
*AMoL*, plate caption.

97 DAP 280, 234.

98 ‘Baby Pioneers Give Birth to a Book’, *Cambridge Evening News*, 19 April 1980, 12; for a ghostwriter as ‘[l]ike a surrogate mother’: Barbara Feinman Todd , Pretend I’m Not Here: How I Worked with Three Newspaper Icons, One Powerful First Lady, and Still Managed to Dig Myself out of the Washington Swamp (New York: Morrow, 2017), 124 .

99 Harris to Edwards, 31 July 1979, EDWS 4/14/1.

100 John Stolls, ‘A Triumph for Life …’, *Coventry Evening Telegraph*, 10 April 1980, Property Supplement, 12.

101 Quita Morgan, ‘A Special Reunion for Baby Louise’, *Evening Post*, 31 March 1980, 4 (clipping in DAP 305); Wendy Cooper, ‘How Two Men Worked a Miracle’, *Birmingham Post*, 31 March 1980 (DAP 304/4); Jean Smith, ‘The Birth of a Breakthrough’, *The Scotsman*, 31 March 1980, 8 (DAP 304/5).

102 Margaret Forster, ‘Once upon a Life’, *Evening Standard*, 1 April 1980, 16; DAP 282/1, 5–6.

103 Br[y]an Silcock, ‘The Conceptual Steps towards Conception’, *Sunday Times*, 20 April 1980, 43. For the dispute over the serialisation: Harold Evans to Harris, 10 December; Harris to Evans, 11 December 1979, EDWS 4/14/2.

104 Karl Sabbagh, ‘First Test-Tube Baby’, *BMJ*, 14 June 1980, 1426–7; *AMoL*, 80.

105 Sabbagh, *op. cit.* (n. 104).

106 Crick to Watson, 13 April 1967, quoted in Watson, *op. cit.* (n. 14), 295; on the magazines: Tracey Loughran , ‘Landscape for a Good Woman’s Weekly: Finding Magazines in Post-war British History and Culture’, in Rachel Ritchie et al. (eds), Women in Magazines: Research, Representation, Production and Consumption (New York: Routledge, 2016), ch. 2.

107 Sue Hercombe, ‘Another Matter of Life’, *The Journal*, 31 March 1980, 6.

108

Gena Corea , The Mother Machine: Reproductive Technologies from Artificial Insemination to Artificial Wombs (London: Women’s Press, 1985), 105–15; Patricia Spallone , Beyond Conception: The New Politics of Reproduction (Basingstoke: Macmillan Education, 1989), 91, 110.

109

Sarah Franklin , Embodied Progress: A Cultural Account of Assisted Conception (London: Routledge, 1997), 226, 228 .

110 Promotion schedules: DAP 307/11; 308/1–2.

111
*Publishers Weekly*, 218 (9) (29 August 1980), 295.

112 Charlotte Gray, ‘Bringing Joy to Proud Parents’, *Ottawa Journal*, 21 June 1980, 43.

113 E.g., Peter Singer and Deane Wells , The Reproduction Revolution: New Ways of Making Babies (Oxford: Oxford University Press, 1984), chs 1–2. The 1981 Sphere paperback carried the straplines ‘The sensational story of the world’s first test-tube baby’ and ‘The miracle of the century’ on front and back cover respectively. There were serialisations in the *Nottingham Evening Post*, *Book Digest* magazine, Toronto *Globe and Mail*, *Vancouver Sun*, *Sydney Morning Herald* and *Straits Times*, and translations into Spanish, Dutch, Japanese and Serbo-Croat: agreements for *A Matter of Life*, PRHA.

114 Fiona Bennett to Jo Watt (Random House), 24 August 2011, with note from Watt, ‘*A Matter of Life*: Reversion paperwork’, PRHA; Robert Edwards and Patrick Steptoe, *A Matter of Life: The Story of IVF – a Medical Breakthrough*, 2nd edn, ed. Ruth Edwards and Andrew Steptoe with Fiona Bennett (Kindle, 2012).

115 Jenny Joy to author, 29 January 2020.

116 Zoom interview with Jenny Joy, 23 September 2024.

117 Bennett to Random House, 24 August 2011, ‘*A Matter of Life*: Reversion paperwork’, PRHA.

118 Joy interview, *op. cit.* (n. 116).

119 Zoom interview with Andrew Steptoe, 11 September 2024.

120 Taylor, *op. cit.* (n. 2); quotations: Thorne, *op. cit.* (n. 3).

121 Gareth Farr’s play *A Child of Science* (London: Hern, 2024) also drew on Johnson and Elder’s research to highlight the story of an unsuccessful volunteer, somewhat exaggerate Purdy’s role and represent Ruth Edwards as a scientist as well as a wife and mother, while more thoroughly distorting dates, places and actions.

122
*The Life Scientific* (BBC Radio 4, https://www.bbc.co.uk/programmes/b015sqc7) has broadcast over 300 interviews since 2011.

